# The Genetics of Thermoregulation in Pigs: A Review

**DOI:** 10.3389/fvets.2021.770480

**Published:** 2021-12-13

**Authors:** Jean-Luc Gourdine, Wendy Mercedes Rauw, Hélène Gilbert, Nausicaa Poullet

**Affiliations:** ^1^URZ, INRAE, Domaine Duclos Prise d'eau, Petit-Bourg, France; ^2^Departamento de Mejora Genética Animal, Instituto Nacional de Investigación y Tecnología Agraria y Alimentaria, INIA-CSIC, Madrid, Spain; ^3^GenPhySE, Université de Toulouse, INRAE, INP, Castanet Tolosan, France

**Keywords:** thermoregulation, pig, heat stress, genetics, selection

## Abstract

Heat stress (HS) affects pig performance, health and welfare, resulting in a financial burden to the pig industry. Pigs have a limited number of functional sweat glands and their thermoregulatory mechanisms used to maintain body temperature, are challenged by HS to maintain body temperature. The genetic selection of genotypes tolerant to HS is a promising long-term (adaptation) option that could be combined with other measures at the production system level. This review summarizes the current knowledge on the genetics of thermoregulation in pigs. It also discusses the different phenotypes that can be used in genetic studies, as well as the variability in thermoregulation between pig breeds and the inheritance of traits related to thermoregulation. This review also considers on-going challenges to face for improving heat tolerance in pigs.

## Introduction

Non-ruminants (pigs and poultry) represent the majority of meat consumed in the world (1). Pork production is expected to increase, despite the need to change production practices due to finite natural resources (2) and the need to decrease meat consumption [particularly beef and dairy cattle products (3)] to reduce GHG emissions (4, 5) (In Europe, the objective of the European Commission is−55% GHG by 2030 compared to 1990). Heat stress (HS) impacts pig performance, health and welfare (6), resulting in a financial burden to the pig industry (7). For instance, in the USA, HS has been estimated to cost from $300 (8) to $900 (9) million annually, depending on the year and method of estimation. Climate change, with concomitant changes in the frequency and magnitude of ambient temperatures and precipitation, may accentuate animal health and welfare problems (6). Furthermore, there is growing evidence (10) that genetic selection has reduced pigs' ability to cope with HS, due to an increase of metabolic heat production with the improvement in reproductive traits and lean tissue growth rate (11, 12) at the expense of adaptive capacities (13). Unlike ruminants, pigs have a limited number of functional sweat glands to facilitate heat loss by evaporation, so their thermoregulatory mechanisms are challenged by HS when trying to maintain body temperature (14). There is a great amount of research that proposes adaptation solutions aimed at reducing the negative effects of climate change on livestock production (3, 15, 16). Several solutions are already available and implemented, such as altering the environmental (cooling options) or feeding management (changes in diet composition and/or distribution) (7). However, some of these adaptation strategies could come at a high cost, both financially and environmentally. The genetic selection of genotypes tolerant to HS is a promising long-term (adaptation) option that could be combined with other measures at the production system level (16, 17). Furthermore, in the framework of agroecology (18, 19), selection must be considered in relation to the food system and the value chain. This implies that we should no longer try to isolate animals from the fluctuations of the environment of production, but rather to favor their capacity to produce and to reproduce in less controlled environments, including in HS conditions (20). The focus of this review is to discuss our current knowledge of the genetics of thermoregulation in pigs. Deepening our understanding of the genetic variability of thermoregulation in pigs and how it can be used to select animals with better heat tolerance is essential to develop strategies to mitigate the negative effects of climate change on pig production.

## The Phenome of Thermoregulation in Pigs

### Thermoregulation in Pigs

**Figure 1:**
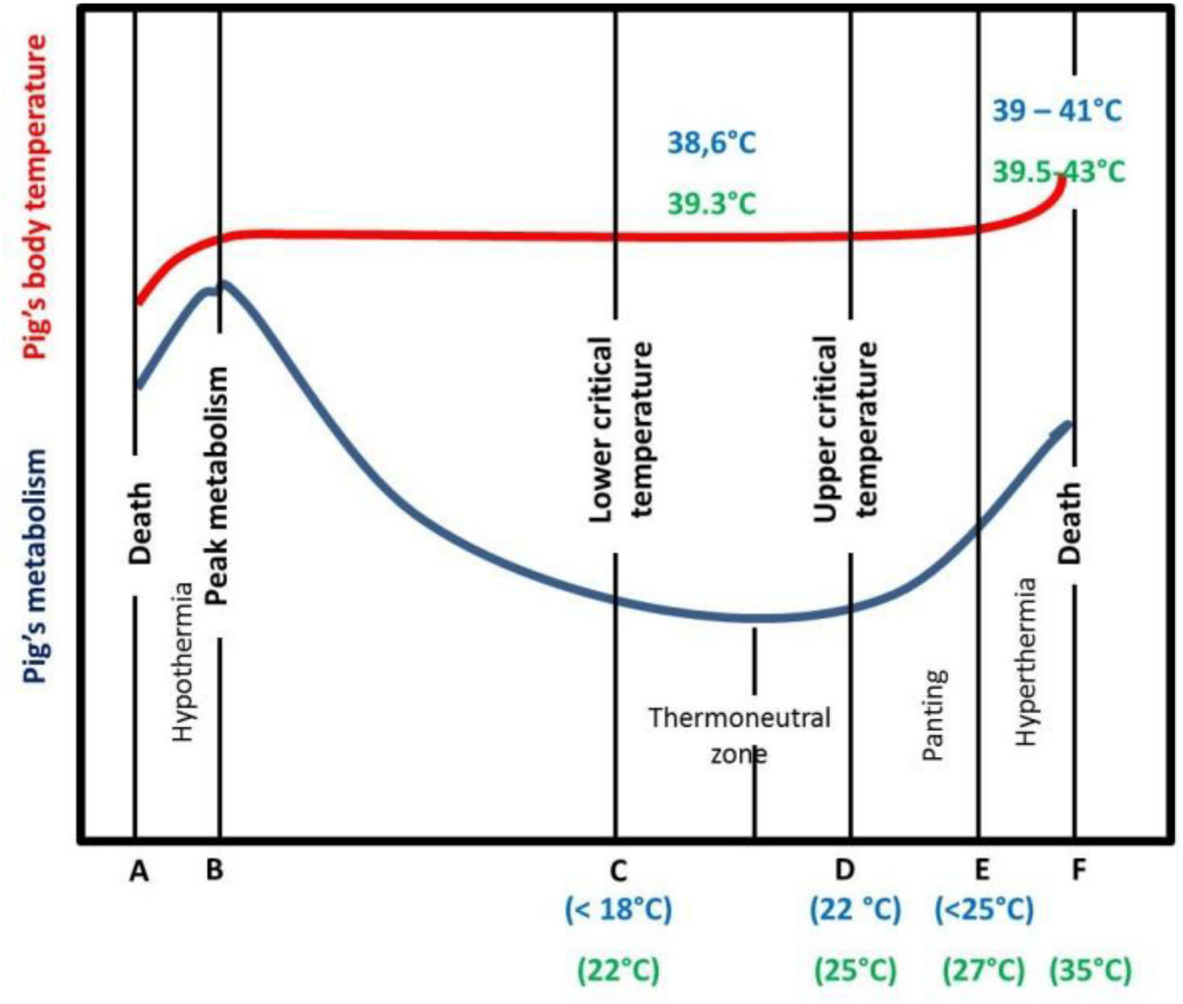
Diagrammatic presentation of the effect of ambient temperature on lactating sow (indicative values in blue) and growing pig (indicative values in green) metabolism and body temperature [adapted from (32)]. The lower critical temperature is the ambient temperature below which pigs must increase heat production to maintain heat balance. The upper critical temperature is the ambient temperature above which pigs must increase heat loss rate to achieve heat balance.

Pigs are homeothermic animals as they can keep deep body temperature relatively constant, within narrow limits, despite a wide variation of the surrounding climatic environment. Thermoregulation is the physiological process allowing the balance between heat production and heat loss mechanisms (21). We assume that from an animal production point of view, physiological HS can be defined as the magnitude of environmental and metabolic loads for which the animal cannot dissipate an adequate quantity of heat to maintain homeostasis with minimal performance losses (22). Figure 1 schematically illustrates the relationship between ambient temperature, heat production and heat loss. Illustrative values of critical and rectal temperatures are also given, based on available data from the literature in lactating sows (23) and in growing pigs (10). These critical temperatures vary greatly, depending on numerous factors such as breed, body weight and composition, diet management, group size, and temperature by humidity interactions (24). Pigs can lose heat by conduction, convection and radiation (sensible heat loss), and by evaporation (latent heat loss). In the thermoneutral zone (from temperatures C to D, Figure 1), pig metabolism (and heat production) is relatively constant. When the ambient temperature increases above D, sensible heat transfer becomes ineffective due to the reduction of the temperature gradient between skin and ambient air. Pigs then rely mainly on evaporative heat loss by increasing respiratory rate to maintain a constant body temperature (25). The ability of the pig to dissipate heat is actually a combination of the effect of ambient temperature and humidity, that can be captured by the temperature humidity index (THI) (26–28). Most studies (27–29) designed to characterize the effect of heat load from the environment on livestock responses have summarized the climatic factors in a THI index. The levels of panting, metabolism, and body core temperature differ between physiological stages and animals. Animals in stages of high metabolic activities (lactation, growth) are typically more susceptible, as well as animals with a low surface/area body weight ratio. Furthermore, there are short-term responses to HS [acclimation or acclimatization (21)] that differ from long-term ones (adaptation). In addition HS varies in duration (short periods of HS, heat waves of few days' duration or chronic HS) and magnitude (moderate, high, or extreme) (30). The improvement of thermoregulation in pigs by genetic selection assumes that there is a genetic component of traits associated with thermoregulation. Hence, if thermoregulation is heritable, animals, breeds, or lines with higher C, D, E, or F values could be selected, resulting in an increased tolerance to HS (31).

### How to Phenotype Thermoregulation in Genetic Studies?

The regulation of body temperature is a complex physiological process involving regulation from the cell to the whole animal, therefore, the phenome of thermoregulation encompasses a large variety of physical and biochemical traits (21). The starting point of genetic studies to perform selection for more heat tolerant animals is to define heritable phenotypes associated with resistance or susceptibility to heat stress. One of the aims of such studies would be to obtain relevant genetic markers or genes for a better genomic evaluation of heat tolerance. So far, the main criterion considered in breeding programs is the maintenance of production performance under HS (33). Other traits directly related to thermoregulation should be considered as potential selection criteria. The main challenges with phenotyping trait indicators of thermoregulation capacities in genetic studies are to accurately define phenotypes. Desirable phenotypes should (i) be biologically relevant, (ii) be technically easy to measure routinely in genetic evaluations schemes at low financial and energetic cost levels, and (iii) fall within the following framework: more non-invasive phenotypic parameters to monitor is desirable, for animal welfare and for the representativeness of the measured phenotypes to the physiological reality of the animal.

As reviewed by Renaudeau et al. (32), several physiological traits (macro-phenotypes and biomarkers) that are directly or indirectly related to heat production, heat loss, and body core temperature can be measured. Rectal temperature is an indicator of body core temperature, which is the result of the whole thermoregulation process. Skin temperatures measured at several sites are indicators of sensible heat loss. More precisely, with rectal, skin and ambient temperatures or THI, a thermal circulation index can be calculated as an indicator of blood flow to the skin to promote sensible heat loss (34). Respiratory rate is an indicator of latent heat dissipation. Several blood metabolites and hormones associated with the HS response have been proposed as potential biomarkers for HS (32, 35, 36). The main biomolecules associated with the HS response in pigs that have been reported in the literature are summarized in Table 1. These studies were largely conducted using commercial breeds, that were more sensitive to HS. The hypothalamic–pituitary–adrenocortical (HPA) axis is one of the most important stress-responsive neuroendocrine systems. The activation of the HPA axis leads to the production of cortisol which is released into circulation and represents one of the principal stress hormones in livestock species (36, 37). Plasma or serum is mostly used to measure cortisol levels (37). However, there is a growing tendency to use cortisol level from saliva (38, 39), which is a non-invasive measure and correlates well with serum levels (40). In pigs, salivary cortisol increases with HS and shows a circadian pattern (39). Thyroid hormones T3 and T4 play a vital role in regulating thermogenesis and are also identified as indicators of the response to HS in livestock species (41, 42). High temperature reduces the levels of plasma thyroid hormones and the T3:T4 ratio (i.e., conversion rate of T4–T3) in pigs (43, 44), suggesting reduced metabolic rate. Another important metabolic regulator and indicator of HS is the amount of circulating non-esterified fatty acids (NEFA). Several studies showed decreased plasma or serum NEFA levels in pigs under HS, suggesting reduced adipose tissue mobilization (44–46). Moreover, heat-stressed animals, despite having lower feed intake, exhibit higher insulin levels. This paradox may be explained by the insulin's role in activating heat shock proteins (HSP) (47). The increase in circulating insulin is correlated with HSP70 expression (45) and both insulin and HSP90 response are required for successful adaptation to HS (43).

**Table 1:** List of traits associated to thermoregulation use in pig studies.

**Traits**	**Tools used to measure**	**Proxies of**	**Invasiveness**
**Functional traits**			
Rectal temperature	Thermometer	Body core temperature	Moderate
Respiratory rate	Observation of flank movement	Latent heat loss	No
Skin temperature	Infrared thermometer	Sensitive heat loss	No
Cortisol	Cotton bud for salivary measure	Stress	Moderate
	Blood sampling	Stress	High
T3/T4 thyroid hormones	Blood sampling	Thermoregulation (through reduced metabolic activity)	Moderate
Non-esterified fatty acids (NEFA)	Blood sampling	Thermoregulation (through reduced lipolysis)	Moderate
Heat-Shock protein HSP70/90 mRNA expression	Tissue sampling (blood, liver, muscle, adipose tissue…)	Heat stress	Moderate to severe
**Production traits**			
Growth rate	Balance	Thermoregulation	No
Feed intake	Automatic feeder	Thermoregulation	No
Feed efficiency	Balance/Automatic feeder	Thermoregulation	No
Physical behavior	Video-recording	Sensitive heat loss	No
Feeding behavior	Automatic feeder/Video-Recording	Body core temperature	No
Drinking behavior	Video-recording	Heat loss	No

The hormonal, cellular and molecular response to HS is therefore complex and is still being unraveled. Moreover, little data is available on the heritability of these biomarkers and their correlation with other HS phenotypes and with production traits, making it difficult to postulate on the most relevant biomarkers to include in genetic studies. Because HS reduces feed intake, it can be difficult to determine whether these responses are the consequences of direct effects of HS, or of indirect effects linked to the reduced feed intake (44, 45, 48, 49). Moreover, most of these measures are obtained from blood sampling methods that are costly in production conditions and that are invasive to animal welfare. Nevertheless, understanding the metabolic and cellular response to HS is essential to eventually find proxies or alternative measurements (such as salivary measures) for these parameters that can be used in genetic studies.

Finally, the variation in traits of economic interest in response to the heat load, such as reproduction (e.g., fertility, prolificity) and production traits (e.g., growth rate, feed intake, and feed efficiency) are indicators of resilience to HS (50, 51). These traits could also be useful to identify the most interesting traits which highlight the biological response to heat. For instance, feed intake is likely to respond faster than body weight to HS, as reducing intake reduces metabolic heat production. Consequently, analysis of feeding behaviors could be a non-invasive and easy-to-measure proxy for body core temperature, as feeding behavior patterns have been shown to change significantly with temperature (52). Under HS, pigs spend less time eating and reduce meal size and duration (53, 54) and feeding rate (52), probably as a way to reduce heat production by decreasing physical and metabolic activity (55). It is important to emphasize that the quantification of criteria to characterize thermoregulation should be done in relation with the fluctuations of the climatic conditions to capture the animal's response to heat load. Several statistical models have been developed to quantify trait changes due to HS: as a slope (56) or as coefficients associated to the broken lines of the curve (22, 57), or as indicators of trait variability (50). For instance, in lactating sows, who are particularly sensitive to heat stress, due to their high metabolic heat production for milk production (58), longitudinal measures of traits associated with thermoregulation during lactation or from gestation to weaning provide more accurate information than a single measure to characterize the best moment for phenotyping both within a day and during the gestation and lactation periods. Carabaño et al. (57) and Gourdine et al. (59) reported that the most discriminating daily period (i.e., maximum range) for thermoregulatory variation is between 04:00 to 07:00 h and 19:00 to 23:00 h, in relation with the hourly feed intake and the circadian rhythm of body core temperature. At the lactation scale, other studies (60) have suggested that the dynamics of rectal temperature was directly related to the kinetic metabolic heat production related to energy and protein intake and milk synthesis. Furthermore, from a meta-analysis (23), a curvilinear relation was found between the increase in average rectal temperature of lactating sows and ambient temperature, with an increase in rectal temperature of 0.07°C per degree of ambient temperature.

## Genetic and Genomic Considerations for Thermoregulation in Pigs

### Is There Variability for Thermoregulation Traits Between Breed or Line?

**Figure 2:**
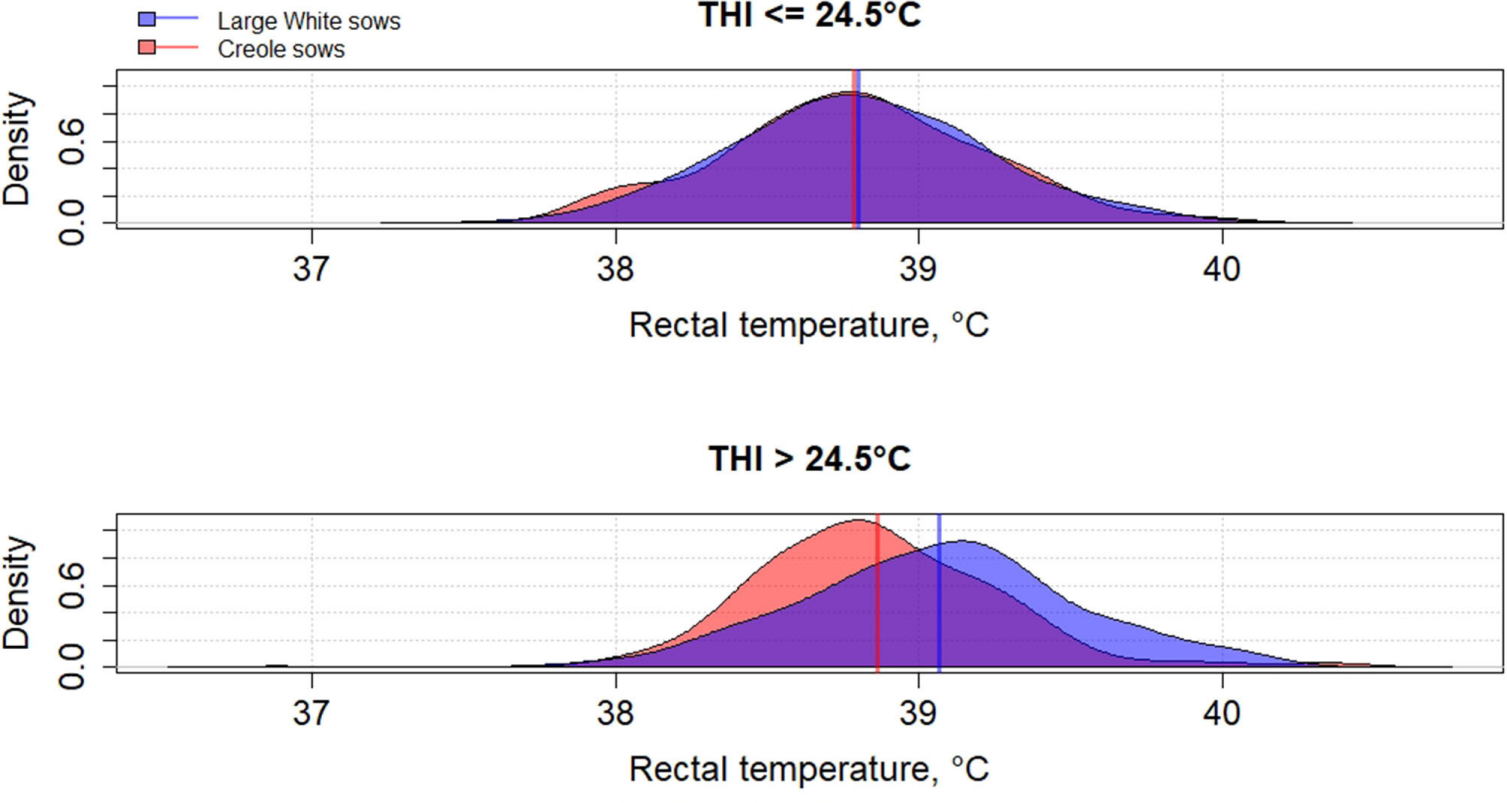
Density distribution of average rectal temperature of Creole and Large White lactating sows according to the average thermal-humidity index (THI) during lactation [adapted from Gourdine et al. (59)].

**Figure 3:**
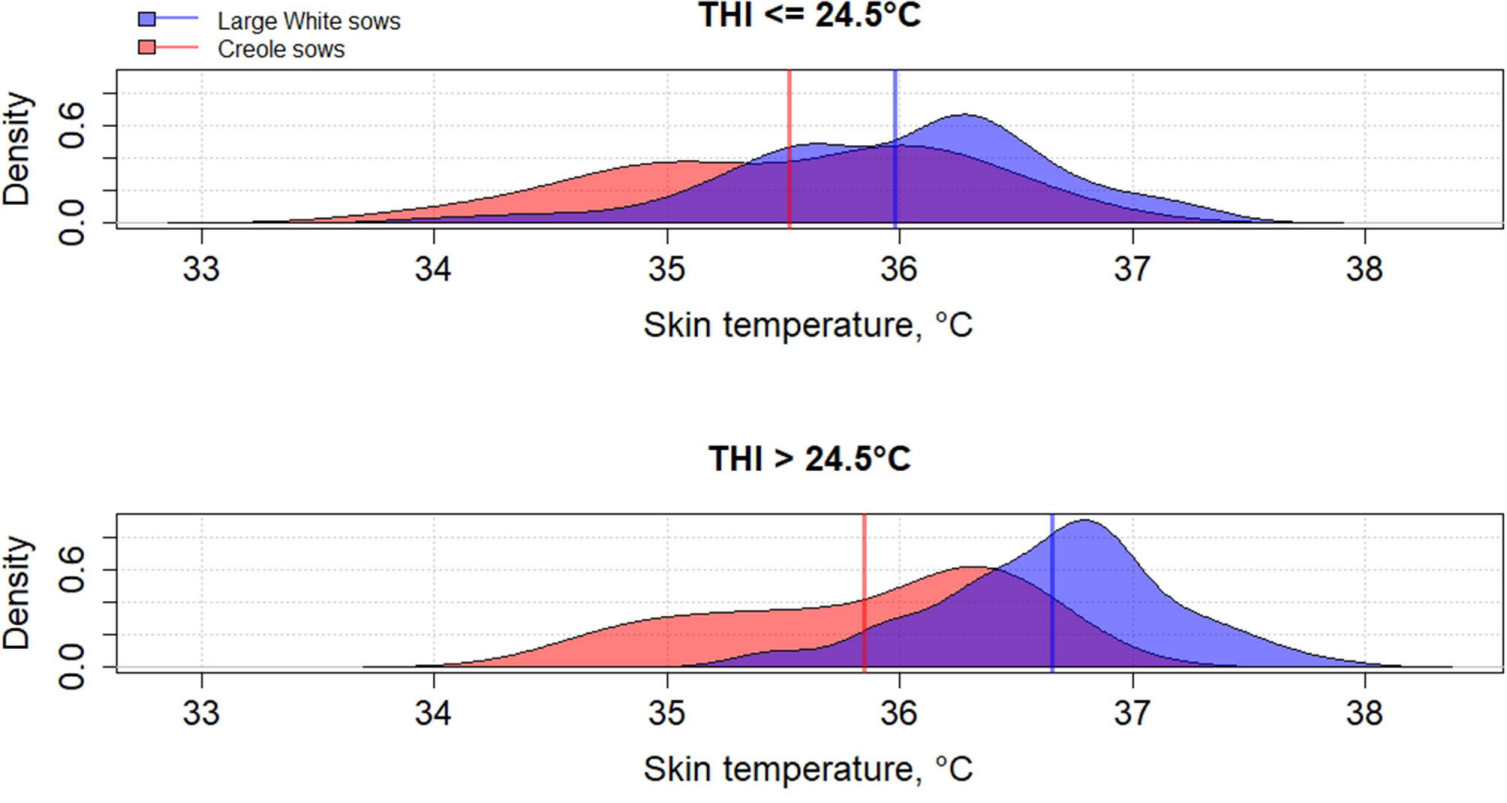
Density distribution of average skin temperature of Creole and Large White lactating sows according to the average thermal-humidity index (THI) during lactation [adapted from Gourdine et al. (59)].

**Figure 4:**
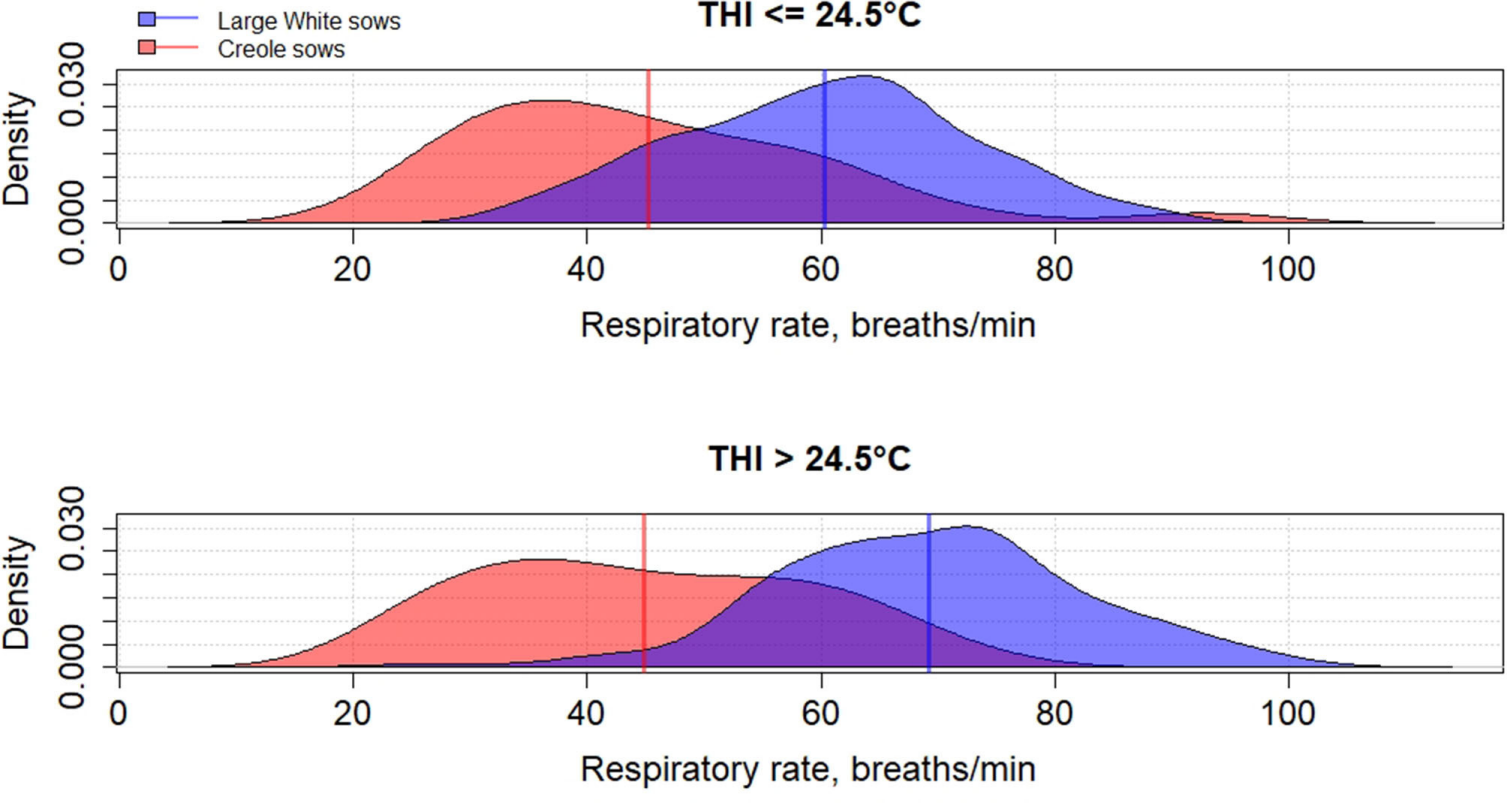
Density distribution of average respiratory rate of Creole and Large White lactating sows according to the average thermal-humidity index (THI) during lactation [adapted from Gourdine et al. (59)].

Genetic variation between breeds in response to HS have been reported in several species such as cattle (61, 62), poultry (63), and pigs (64–66). To the best of our knowledge, little has been published on the differences in heat tolerance between tropical and temperate pig breeds, despite most of pig breeds being from tropical and subtropical areas (67). In these areas, microevolution has promoted the emergence of breeds with a high ability to cope with HS but these breeds remain poorly characterized (68). The physiological adaptation to HS of tropical breeds could be partly explained by a lower metabolic heat production due to a lower productive potential of tropical breeds than commercial breeds, but also by a higher ability of some breeds to dissipate heat than other breeds, probably related to favorable alleles. Figures 2–**4** illustrate the variability of thermoregulatory responses to HS between a tropical local Creole breed (local tropical breed) and a Large White breed (European commercial breed) in lactating sows. Within the same production environment, high average THI during lactation caused greater increase of rectal temperature (Figure 2), skin temperature (Figure 3) and respiratory rate (Figure 4) in Large White than in Creole sows, suggesting better physiological adaptation of Creole sows to HS. Moreover, higher variation within Creole sows than within Large White sows illustrates the high variability of an unselected breed compared to a selected one. In growing pigs, studies comparing the same breeds (Large White vs. Creole) have shown that the effect of HS on thermoregulatory responses were higher in Large White than Creole pigs, whether under chronic HS, such as seasonal effects in indoors (54) or outdoors (69), or under short-term HS (70), showing the existence of breeds with higher critical temperatures values (e.g., in the tropical conditions of Guadeloupe, the upper critical THI for Large White sow is around 24.5°C and the corresponding values for Creole sow is >24.5°C) (59). To our knowledge, there is no data in the literature about the hormonal and metabolic response of tropical breeds to HS, and it is therefore difficult to assess how the potential biomarkers mentioned above predict how the physiological mechanisms have evolved under HS in these breeds. However, several studies showed that cortisol levels are highly genetically variable, even in highly selected pig breeds (71, 72). It would be of particular interest to characterize neuro-endocrine (cortisol), metabolic (thyroid hormones, NEFA) and cellular and molecular (HSP proteins) responses to HS in heat tolerant breeds to evaluate the relevance of these potential biomarkers for selection of heat tolerant animals.

When comparing feeding behavior of Creole and Large White pigs under HS, both breeds had similar daily feed intake but different feeding behavior patterns, with fewer but larger meals for the Creole associated with a lower feeding rate (54). Differences in feeding behavior in response to HS have been observed in other breeds (73) and support the idea of using feeding behavior as a proxy for thermoregulation. In this context, the genetic variation responsible for the natural thermotolerance of tropical local pig breeds could be used for genetic improvement for heat tolerance of international commercial pig breeds. To our knowledge, only a few studies have dealt with this topic (50), despite the widespread use of crossbreeding schemes in the pig industry. Crossbreeding with well-heat-adapted but less productive breeds (which partly explain their better tolerance to HS) might be financially less profitable in many contexts such as large intensive operations, due to the payment grid and production costs. Indeed, crossbred pigs with tropical local pig genetics would grow slower and fatter with heterogeneous groups of pigs to manage (74), which often means penalties from the abattoir. However, when and if knowledge allows it, introgression of favorable alleles to HS from tropical pig breeds into commercial pig lines could be a promising technique to improve heat tolerance.

### Are Thermoregulation Traits Heritable?

In contrast to production traits, the inheritance of traits associated to thermoregulation has been poorly described in the literature. In species such as cattle (75–77), rectal temperature is the main thermoregulation trait for which heritability was accurately estimated (Table 2). In pigs, estimation of genetic parameters of thermoregulation traits rarely exist. For instance, to our best knowledge, few studies have estimated heritabilities of rectal temperature in piglets (78), in growing pigs (79) and in sows (22, 80), essentially pointing out low to moderate genetic bases for body and skin temperatures. However, thermoregulation is a physiological homeostatic process, meaning that the steady state of the internal temperature is maintained. That raises the question of which trait should be genetically improved for a better thermoregulation. Genetic improvement of body core temperature should not target the average body core temperature (for instance increasing the mean from 38.7 to 39.7°C), but rather focus on reducing its variance, with the aim to decrease the number of heat-stressed pigs. At the animal level, that corresponds to animals with a better regulation of body temperature during heat stress.

**Table 2:** Heritabilities (h^2^ ± SE) of body temperature and respiratory rate in different livestock species.

**References**	**Species**	**Breed or line**	**Physiological stage**	***N*. records (*N*. animals)^**a**^**	**Trait^**b**^**	**h^2^ ± SE**	**Conditions^**c**^**
Morris et al. (81)	Bovine	Charolais, Murray gray, Simmental, Red devon	Steers and heifers	3,839 (611)	RT	0.19 ± 0.09	In Te Awamutu in New Zealand
Lemos and Lôba (82)	Bovine	Pitangueiras	Not available	125–275 per generation (5 generations)	RT	0.15 ± 0.09 to 0.27 ± 0.12	Measured in the morning (04:00 to 08:00) in the tropical conditions of Pitangueiras in Brazil
					RT	0.17 ± 0.10 to 0.31 ± 0.13	Measured in the afternoon (12:00 to 18:00)
					ΔRT	0.16 ± 0.16 to 0.27 ± 0.11	Difference between RT measured in the afternoon and in the morning
Mackinnon et al. (83)	Bovine	Zebu × *Bos Taurus* crosses	Post-weaning	7,174 (1,341)	RT	0.19 ± 0.02	Animals left unshaded and without food for 3 h during the highest heat stress (in August after weaning and the following May) in Queensland in Australia
Burrow (84)	Bovine	AX and AXBX beef cattle	Birth to 18 months of age	11,930 (2,403)	RT	0.18 ± Not available	RT recorded 4 and 7 times per animals when T was > 30°C
Prayaga et al. (85)	Bovine	Brahman and Tropical composite beef cattle	Heifer at 400 days of age	1,065	RT	0.21 ± 0.09	RT was measured during summer months when the ambient temperature was >30°C, in Queensland, in Australia
Dikmen et al. (86)	Bovine	Holstein	Lactating cows	1,695	RT	0.17 ± 0.13	Afternoon RT (15:00–17:00) during the summer in Florida, in USA
Riley et al. (77)	Bovine	Angus, Brahman, Criollo Romosinuamo	Cow–calf	3,396 (2,200)	RT	0.19 ± 0.03	Subtropical summer conditions in Florida in USA
Porto-Neto et al. (87)	Bovine	Brahman	Post-weaning	2,112	RT	0.22 ± Not available	Repeated RT measures collected at various post-weaning ages in Northern Australia
		Tropical composite		2,533	RT	0.14 ± Not available	
Davila et al. (76)	Bovine	Brahman-Angus	Heifer	334	VT	0.32 ± 0.18	At low THI (68–70) in Florida in USA^c^
				334	VT	0.26 ± 0.16	At high THI (37, 80, 88)
Otto et al. (89)	Bovine	Gir × Holstein F2	Post-weaning	653 (341)	ΔRT	0.13 ± 0.08	Animals were housed in a heat chamber in Embrapa in Brazil. The ΔRT is the difference between RT measured 6 h after the heat chamber reached T = 42°C and RH = 60%, and after 12 h of adaptation to the heat chamber at T = 22°C and RH = 50 %.
Luo et al. (75)	Bovine	Holstein	Lactating cows	59,265 (13,592)	RT	0.06 ± 0.01	RT and RR were measured during summer period in Beijing, in China
				30,290 (13,592)	RR	0.04 ± 0.01	
Taouis et al. (90)	Poultry	Hybrid broiler	Birth to 7 days of age	161	ΔRT	0.36 ± 0.18	Early-age thermal conditioning at 5 d of age exposed at 40°C for 24 h
Van Goor et al. (91)	Poultry	Generations F18 and F19 of a broiler (heat-susceptible) × Fayoumi (heat-resistant) intercross line	20 days of age	631	RT	0.11 ± 0.06	Climatic chambers at 22°C from 17 to 22 days of age; at 35°C for 7 h per day and remained at 25 °C at all other: from 22 to 28 of days age. Cloacal body temperatures were measured on days of age 20, 22, and 28
			22 days of age	631	RT	0.10 ± 0.06	
			28 days of age	631	RT	0.10 ± 0.06	
			From 20 to 28 days of age	631	ΔRT	0.03 ± 0.04	Differential of cloacal body temperature measured on days of age 28 and 20
Kaushik et al. (92)	Goat	Jamunapari breed	Kids: 6–9 month and adults: 2 to 3 year	695	RT	0.36 ± 0.12	During May-June (average T: 45.9 ± 0.5 °C; average RH: 28.2 ± 1.8%) at Mathura, in India. RT recorded at the highest temperature of the day (13:30 to 14:30)
				617	RT	014 ± 0.10	During December-January (average T: 22.5 ± 0.6°C; average RH: 83.1 ± 2.1%) at Mathura, in India. RT recorded at the lowest temperature of the day (09:00–10:00)
Varona et al. (78)	Pig Pig	Iberian × Meishan Iberian × Meishan	Newborn piglets Newborn piglets	415	RT	0.10-0.55	RT at birth recorded in Lleida, in Spain
				395	RT	0.02-0.58	RT 60 min after birth
Gourdine et al. (59)	Pig	Large White	Lactating sows	842 (220)	RT	0.35 ± 0.09	Average RT, CT and RR during lactation, measured in tropical humid conditions of Petit-Bourg, in Guadeloupe (average T: 24.7 ± 1.3°C; average RH: 89.3 ± 5.6 %)
				245 (126)	CT	0.34 ± 0.12	
				403 (151)	RR	0.39 ± 0.13	
Kim et al. (80)	Pig	Crossbred PIC maternal × Duroc	Pre-pubertal gilts	214	ΔRT	0.49 ± not available	Gilts were previously in thermoneutral conditions during 96 h (average T: 21.9 ± 0.5°C, average RH: 62 ± 13%) and after they were submitted a 24 h HS challenge (average T: 29.7 ± 1.3°C; average RH: 49 ± 8%). ΔRT and ΔRR are the difference between values during HS and thermoneutral conditions
					ΔRR	0.39 ± not available	
			Post-pubertal gilts	100	ΔRT	0.83 ± not available	Gilts were preliminary selected based on their ability or inability to maintain a minimal RT during the 24 h HS challenge. TR and RR were collected in post-pubertal during thermoneutral conditions (20°C). ΔRT is the difference between values during HS and thermoneutral conditions
Gourdine et al. (79)	Pig	Crossbred ¾ large white × ¼ creole breed	Growing pigs at 19 weeks of age	630	RT	0.04 ± 0.05 to 0.13 ± 0.07	Growing pigs in the temperate condition of Charentes, in France (T between 20.5 and 27.7°C; RH between 46.2 and 76.3%)
			Growing pigs at 23 weeks of age	627	RT	0.07 ± 0.06 to 0.34 ± 0.12	
			Growing pigs at 19 weeks of age	663	RT	0.12 ± 0.07 to 0.17 ± 0.07	Growing pigs in the tropical humid condition of Petit-Bourg, in Guadeloupe (T between 22.2 and 228.9°C; RH between 75.3 and 93.6%)
			Growing pigs at 23 weeks of age	663	RT	0.08 ± 0.06 to 0.10 ± 0.05	

a *The number of animals measured was in parenthesis when the number of observations did not correspond to the number of animals*;

b *RT, rectal temperature; VT, vaginal temperature; RR, respiratory rate; CT, cutaneous temperature*;

c *T, ambient temperature; RH, relative humidity; THI was calculated as THI = (1.8 × T + 32)–[(0.55–0.0055 × RH) × (1.8 × T−26)]*.

Similarly to thermoregulation traits, little data is available on the genetic parameters of potential biomarkers for HS in pigs. In pigs, cortisol levels were found to be highly heritable (h^2^ = 0.68) (93) and in dairy cows, NEFA was found to be moderately heritable [0.08–0.35 (94, 95)]. To our knowledge, no studies have investigated the genetic correlations between these metabolic markers and performance traits in pigs. However, higher cortisol has been shown to have positive effects on traits related to robustness and adaptation, while having negative effects on production traits (growth rate, fat/lean ratio) (37). Foury et al. (72) found that cortisol and carcass lean content were phenotypically negatively correlated (−0.46), so that ~21% of variation in fatness across breeds could be explained by variation in cortisol. These results need to be confirmed by genetic studies but suggest that it might be possible to select for cortisol levels allowing better heat tolerance without compromising production traits.

Using feeding behavior as a proxy for thermoregulation, a pangenomic study has been performed (73) to detect genomic variants associated with changes in feeding behavior under HS. The authors found that heritabilities for differences in feeding activity were low to moderate, suggesting that heat tolerance is heritable, and suggested some candidates genes, such as *DPYSL2* and *ADRA1A*, and biological pathways (e.g., immune function) to explain the detected associations.

As reviewed by Renaudeau et al. (24), there is a moderate to strong negative phenotypic correlation between rectal temperature and production traits in many species, suggesting that thermoregulation and production traits may be partly governed by separate genomic loci. However, estimates of the association between these traits often had large standard errors due to the limited number of animals in most experimental designs and they depend on the conditions of recording (i.e., if HS occurs or not, and how HS occurs). In acute HS (e.g., 24 h of severe HS of 29.7°C), some studies have shown little or no association between HS and sow feed intake or changes in body weight (80). Like other complex traits, large numbers of genes are probably involved in thermoregulation, and the genetic correlations with other economically important traits should be quantified to investigate possible antagonisms or favorable relationships that should be accounted for in a selection index to improve heat tolerance. If loci involved in variation of production and thermoregulation traits are in linkage disequilibrium, it can be expected that favorable alleles for production traits are associated with unfavorable alleles for thermoregulation, and trade-offs will have to be found between production and regulation capacity during HS. Few studies provide “omics” information for thermoregulation in pigs. In cattle (89, 96) and in poultry (97, 98), the slick hair gene and the naked neck gene, respectively, are used by introgression or crossbreeding to improve the thermotolerance of breeds. QTL have been found associated to body temperature in the Japanese quail (98), in poultry (99), and in cattle (91). To the best of our knowledge, quantitative trait loci (QTL) associated with thermoregulation traits in response to HS were only reported in the studies of Kim et al. (80) in gilts and Riquet et al. (100) in growing pigs. These studies, either with genome-wide association analysis or linkage analysis, have detected a small number of loci (<100) with very small effect on the variability of thermoregulation traits. Increasing the sample size of the pig population to be measured and to be genotyped is therefore necessary, to detect a larger number of SNPs of interest and to decrease the confidence interval of the SNPs' position (80). The sequencing technologies show great improvement and it should be possible in the near future to directly incorporate the causal polymorphisms of the variability of thermoregulation traits instead of genomic markers (33). However, our current knowledge about the genomic variability of thermoregulation traits in pig is limited, and further research is needed.

## Challenges to Face

As pointed out by several authors, the analysis of high-throughput phenotypic and genomic data to address issues related to HS, and more generally to health and animal welfare, requires the development of new methods and technologies capable of integrating diverse, heterogeneous, and large-scale data. In this context, the advent of new technologies, omic tools (101, 102) (including genomic, epigenomic, transcriptomic, metabolomic, microbiome information, and genome editing), but also Artificial Intelligence (AI) approaches (such as deep learning, machine learning, etc.) offer new and very promising avenues for analyses to address HS complex problems.

To the best of our knowledge, there is no pig breeding program including traits associated with thermoregulation. Nevertheless, it is likely that international pig breeding companies take advantage of their presence in contrasting areas, from the Northern Europe to South America, to select pigs according to the environment of production, based on the breeding values of production and reproduction traits. Implementation of traits directly associated with thermoregulation in a conventional pig breeding program is not straightforward, firstly due to the difficulty of defining heat tolerance directly in terms of measurable traits (50), secondly due to the difficulty to measure these appropriate phenotypes routinely and technically easily under HS conditions, and thirdly as far we know due to the lack of quantification of economic weights of traits associated to thermoregulation. Furthermore, the choice of breeding approaches will have to deal with the biological antagonism between production traits and thermoregulation traits (24), i.e., probably leading to reduced genetic gains on the current breeding objectives. Finally, substantial differences can be observed between pig's performance in production environments and that in selection environment, which is referred to as genotype by environment interactions (G × E) (103). Therefore, breeding programs should also consider these interactions as they can be a source of inefficiency (104, 105) to transfer the genetic progress to production farms. For instance, the best pigs for a criterion assessed in a temperate environment would not necessary be the best for the same criterion assessed in tropical conditions. To our knowledge, there are very few studies reporting G × E interactions in relation to thermoregulation or HS in pigs (65, 79), compared to available studies in broiler chickens (63), dairy cattle (62), or beef cattle (106, 107), but they show substantial G × E interactions for some production traits. This may be due to the lack of known genetic relationships between animals reared under contrasted climatic conditions and also due to the absence of a significant amount of data to infer the existence and level of G × E interactions.

In summary, the success of selection for improved response to HS may depend on interrelated factors: (i) the extent of G × E interactions; (ii) the level of antagonisms between genes involved in thermoregulation and production biological pathways, (iii) the definition of a heat tolerance index, and (iv) the ability to collect phenotypic and genomic information on a large scale in the appropriate environmental conditions.

There is no doubt that genomic innovations and precision selection (cisgenesis, transgenesis, genome editing) will be mobilized for future genetic studies on the thermoregulation of pigs (33). These studies cannot be disconnected from the complex and systemic issues related to global change and its impacts on pig production (such as the availability of crops for pig feeding, the emergence of new disease or pathogen vectors in certain regions) (108), the ethical issues related to the use of new breeding techniques, and societal questions on animal welfare and research priorities (33).

## Author Contributions

J-LG, WR, HG, and NP assisted in the conception of the study and contributed to manuscript revision, read, and approved the submitted version. All authors contributed to the article and approved the submitted version.

## Conflict of Interest

The authors declare that the research was conducted in the absence of any commercial or financial relationships that could be construed as a potential conflict of interest.

## Publisher's Note

All claims expressed in this article are solely those of the authors and do not necessarily represent those of their affiliated organizations, or those of the publisher, the editors and the reviewers. Any product that may be evaluated in this article, or claim that may be made by its manufacturer, is not guaranteed or endorsed by the publisher.
